# Transplantation of Neuronal-Primed Human Bone Marrow Mesenchymal Stem Cells in Hemiparkinsonian Rodents

**DOI:** 10.1371/journal.pone.0019025

**Published:** 2011-05-23

**Authors:** Melissa L. M. Khoo, Helen Tao, Adrian C. B. Meedeniya, Alan Mackay-Sim, David D. F. Ma

**Affiliations:** 1 Blood Stem Cells and Cancer Research, St Vincent's Centre for Applied Medical Research, Sydney, New South Wales, Australia, and The University of New South Wales, Sydney, New South Wales, Australia; 2 National Centre for Adult Stem Cell Research, Eskitis Institute for Cell and Molecular Therapies, Griffith University, Brisbane, Queensland, Australia; Universidade Federal do Rio de Janeiro, Brazil

## Abstract

Bone marrow-derived human mesenchymal stem cells (hMSCs) have shown promise in *in vitro* neuronal differentiation and in cellular therapy for neurodegenerative disorders, including Parkinson' disease. However, the effects of intracerebral transplantation are not well defined, and studies do not agreed on the optimal neuronal differentiation method. Here, we investigated three growth factor-based neuronal differentiation procedures (using FGF-2/EGF/PDGF/SHH/FGF-8/GDNF), and found all to be capable of eliciting an immature neural phenotype, in terms of cell morphology and gene/protein expression. The neuronal-priming (FGF-2/EGF) method induced neurosphere-like formation and the highest *NES* and *NR4A2* expression by hMSCs. Transplantation of undifferentiated and neuronal-primed hMSCs into the striatum and substantia nigra of 6-OHDA-lesioned hemiparkinsonian rats revealed transient graft survival of 7 days, despite the reported immunosuppressive properties of MSCs and cyclosporine-immunosuppression of rats. Neither differentiation of hMSCs nor induction of host neurogenesis was observed at injection sites, and hMSCs continued producing mesodermal fibronectin. Strategies for improving engraftment and differentiation post-transplantation, such as prior *in vitro* neuronal-priming, nigral and striatal grafting, and co-transplantation of olfactory ensheathing cells that promote neural regeneration, were unable to provide advantages. Innate inflammatory responses (Iba-1-positive microglia/macrophage and GFAP-positive astrocyte activation and accumulation) were detected around grafts within 7 days. Our findings indicate that growth factor-based methods allow hMSC differentiation toward immature neuronal-like cells, and contrary to previous reports, only transient survival and engraftment of hMSCs occurs following transplantation in immunosuppressed hemiparkinsonian rats. In addition, suppression of host innate inflammatory responses may be a key factor for improving hMSC survival and engraftment.

## Introduction

Cellular transplantation is thought to hold great potential for the treatment of Parkinson' disease, since dopaminergic neurons are selectively lost from the substantia nigra (SN) [1], [2]. In the search for a renewable source of dopamine-producing cells, human fetal brain tissue [3], [4], embryonic stem cells (SCs) [5]–[8], and neural SCs/progenitors [9]–[11] have been investigated. Animal studies have yielded encouraging findings including graft survival, dopamine production and alleviation of motor deficits. Furthermore, recent clinical trials examining human fetal mesencephalic tissue transplantation into Parkinson' disease patients have proven more optimistic than in the past, with most transplants displaying functional activity for at least a decade [12]–[14].

Neuronal differentiation of mesenchymal stem cells (MSCs; also marrow stromal cells) has been achieved through a wide range of approaches involving growth factors/signaling molecules, chemicals, or a combination of both [15]–[25]. The validity of *in vitro* MSC neuronal differentiation, particularly with chemical-based methods, has recently been shrouded in controversy, with findings that the rapid effects caused by chemical exposure resulted from culture artifacts due to cellular toxicity, cell shrinkage and actin cytoskeleton disruption [21], [26], [27]. Nevertheless, growth factor-based neural differentiation has yielded promising results, with an earlier study by our group demonstrating active and dynamic responses to growth factor-induction, including the outgrowth and motility of cellular extensions [28], whilst others have also shown the acquisition of functional properties [29]–[32].

A number of studies have examined the ability of MSCs to differentiate into dopamine-producing cells, re-innervate the striatum, and ameliorate behavioral deficits in Parkinsonian models. Varying degrees of success have been achieved *in vitro*, including dopaminergic marker expression, electrophysiological-activity, and/or dopamine secretion in response to depolarization [20], [22], [23], [32]–[35]. In addition, engraftment and functional improvement were demonstrated following transplantation of undifferentiated [36], [37] and differentiated MSCs [33], [35] in hemiparkinsonian rodents. However, only relatively low efficiencies of dopaminergic differentiation (11%–41%) were achieved, and comparisons between the varying methods have not been performed, resulting in difficulties with identifying the optimal methodology.

Olfactory ensheathing cells (OECs) have been shown to possess unique properties advantageous for autologous transplantation in the nervous system [38]–[42]. OECs are easily obtained through nasal olfactory mucosa biopsy [43]–[45], and transplantation of OECs promotes axonal regeneration and behavioral recovery in spinal cord injury models [46], [47]. In addition, co-transplantation with fetal ventral mesencephalic cells in hemiparkinsonian rats resulted in significant restoration of amphetamine-induced rotational behavior, dopamine levels, and tyrosine hydroxylase (TH) immunoreactivity [48], [49]. Given the success of OEC transplantation in neurological disease models, co-transplantation of OECs may be beneficial for the engraftment and differentiation of MSCs in neurological environments.

At present, a range of approaches have been implemented in examining MSC dopaminergic differentiation and transplantation in Parkinsonian models. Conflicting results have been obtained and studies are not agreed on the optimal methodology. As a result, pertinent issues remain to be resolved, including the optimal method for inducing a dopaminergic phenotype from MSCs, engraftment and survival capabilities of MSCs, optimal sites for transplantation, potential immunological responses to MSC grafts, and whether differentiation prior to transplantation provides engraftment advantages. We addressed these issues by, firstly, examining the generation of dopaminergic neuronal-like cells from hMSCs through comparing three growth factor-based *in vitro* methods, including a single-stage neuronal differentiation (SingleND) procedure [21], a recently published single-stage dopaminergic neuronal differentiation (SingleDA) method [32], and a multiple-stage dopaminergic neuronal differentiation (MultiDA) method that involves sequential stimulation with growth factors important in midbrain dopaminergic neuron development. Secondly, undifferentiated and neuronal-primed hMSCs were transplanted into immunosuppressed hemiparkinsonian rats to investigate graft survival and differentiation. Cells were injected into the striatum, as this is the region requiring dopamine provision and the site most commonly targeted in cellular therapies for Parkinson' disease. We also injected hMSCs into the SN, since midbrain dopaminergic neurons develop in this region. Thirdly, OECs were co-transplanted to evaluate whether advantages or synergistic effects could be provided. The neurotrophic and immunomodulatory effects of hMSCs on host cells were also examined, since these mechanisms may play a role in MSC-mediated improvement or restoration of neural deficits.

## Materials and Methods

### Ethics Statement

All research involving human participants was performed with approval by the Human Research Ethics Committee of our Institutes (St Vincent' Hospital Sydney, Griffith University, and Brisbane Private Hospital), and with written informed consent obtained.

Animal studies were performed under approval from the Animal Ethics Committee of Griffith University (GU Ref No: SCE/06/05/AEC), and in strict accordance with the Australian Code of Practice for the Care and Use of Animals for Scientific Purposes. All surgery was performed under anesthesia, and all efforts were made to minimize suffering. Animals were housed in pairs with 12-hour light/dark cycle and food/water *ad libitum*.

### Isolation and Expansion of hMSCs

Bone marrow (1–2 mL) was collected into sodium heparin tubes from the posterior iliac crest of hematologically normal donors, and hMSCs were isolated as described [28], using RosetteSep tetrameric antibody-mediated negative selection, density gradient centrifugation, and plastic-adherence. Cells were cultured in expansion medium (adapted from [50]–[52]): 60% DMEM-Low Glucose (Gibco Invitrogen, CA, USA), 40% MCDB-201 medium, 1x insulin-transferrin-selenium, 1x linoleic-acid bovine serum albumin, 10^−9^ M dexamethasone, 10^−4^ M ascorbic acid 2-phosphate (AAP; Sigma-Aldrich, MO, USA), 100 U/mL penicillin, 100 µg/mL streptomycin (Gibco) and 10% heat-inactivated fetal bovine serum (Thermo Trace) at 37°C, 5% CO_2_ and 20% O_2_. Cells were subcultured weekly with 0.05% trypsin/0.53 mM EDTA (Gibco), thoroughly washed and re-plated at 2×10^3^ cells/cm^2^. Population doubling and cell viability were recorded at each passage (P). Flow cytometric analysis and mesodermal differentiation assays were performed, as described [28].

### Neuronal Lineage Differentiation

**Figure 1:**
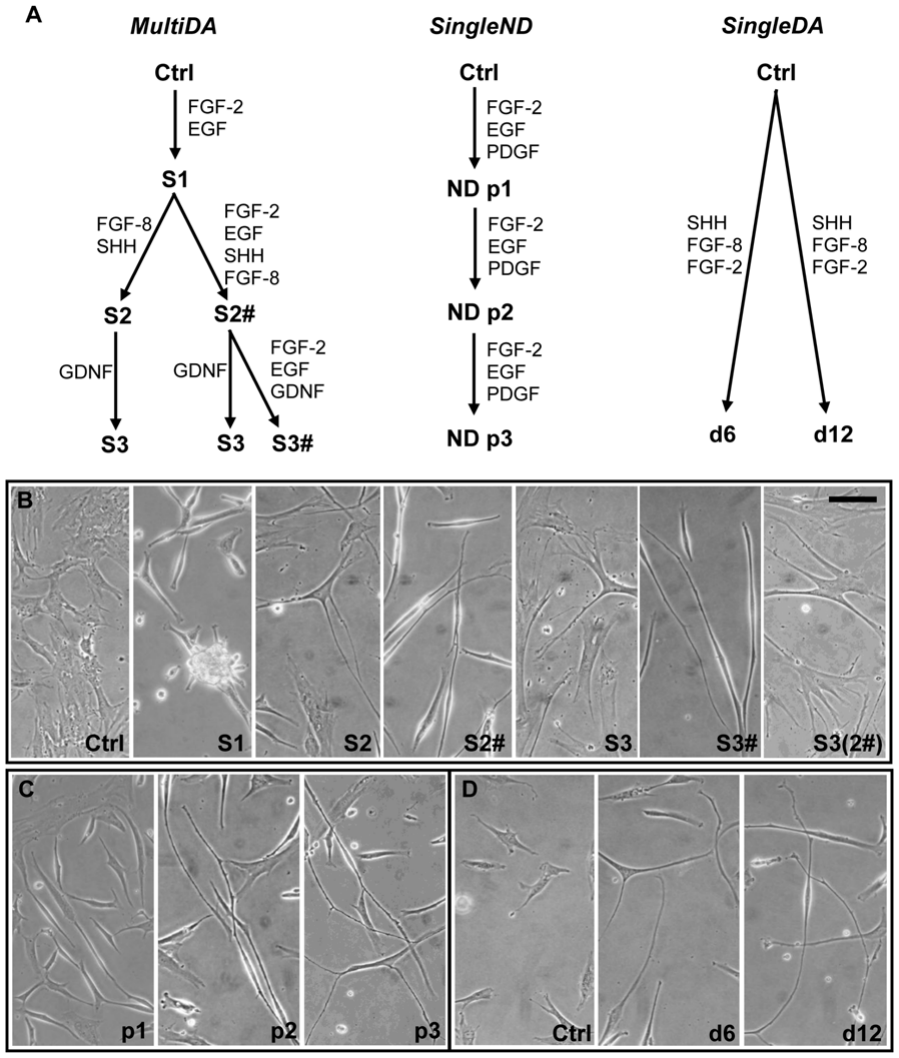
Growth factor-induced neuronal differentiation of hMSCs. (A) Diagram of multiple-stage dopaminergic neuronal differentiation (MultiDA), single-stage neuronal differentiation (SingleND), and single-step dopaminergic neuronal differentiation (SingleDA) [32] methods. (B) Morphology of hMSCs during the MultiDA procedure. Images were captured at the end of each stage (1-week) of sequential growth factor treatment. Representative phase-contrast images depicting hMSCs: prior to treatment (Ctrl; d0); after S1 (d7), S2 (d14), S2# (d14), S3 (d21), S3# (d21), and S3(2#) (d21; S1 growth factors removed after S2) of differentiation (*n* = 3). (C) Morphology of hMSCs during the SingleND procedure. Representative phase-contrast images of hMSCs after: p1 (1 week), p2 (2 weeks), and p3 (3 weeks) (*n* = 3). (D) Morphology of hMSCs during the SingleDA procedure. Representative phase-contrast images of hMSCs: prior to treatment (Ctrl; d0), and d6 and d12 post-treatment (*n* = 3). Ctrl, control undifferentiated hMSCs; S, stage; #, growth factors from S1 included; ND, neuronal differentiation; p, passage; d, day. Scale bar: 100 µm.

Human MSC cultures in exponential growth phase (P4–6) were exposed to three neuronal differentiation procedures (*n* = 3). For SingleND and MultiDA methods: growth factors were replenished daily; medium changed every 2-days; cells were subcultured weekly (trypsin/EDTA for 2-min) and harvested for characterization at 1-, 2- and 3-weeks. Figure 1A summarizes these methods.

#### Single-Stage Neuronal Differentiation (SingleND)

The SingleND method was performed as reported [21]. Briefly, hMSCs were plated at 6.7×10^3^ cells/cm^2^ in fibronectin-coated (Gibco) cultureware (Falcon/Becton Dickinson Labware, NJ, USA) in medium containing DMEM/nutrient mixture F12, 1x N-2 supplement, penicillin/streptomycin (all from Gibco), supplemented with 10 ng/mL Fibroblast Growth Factor (FGF)-2, 10 ng/mL Epidermal Growth Factor (EGF) and 1 ng/mL Platelet-Derived Growth Factor (PDGF)(R&D Systems, MN, USA).

#### Multiple-Stage Dopaminergic Neuronal Differentiation (MultiDA)

Cultureware was pre-coated with 2 µ/cm^2^ Poly-L-ornithine (Sigma-Aldrich) and 1 µg/cm^2^ Mouse Laminin (BD Biosciences, MA, USA), and hMSCs seeded at 6.0×10^3^ cells/cm^2^ in differentiation medium containing DMEM/nutrient mixture F12 (without HEPES), 1x N-2 supplement, and penicillin/streptomycin. Stage-1 medium was supplemented with 10 ng/mL FGF-2 and 10 ng/mL EGF, to prime hMSCs towards a neural fate. Stage-2 medium contained 100 ng/mL Sonic Hedgehog (SHH) (R&D Systems), 10 ng/mL FGF-8 (human) (PeproTech, Inc, NJ, USA) and 200 µM AAP, for initiating midbrain specification. Stage-3 medium contained 50 ng/mL Glial-Derived Neurotrophic Factor (GDNF) (R&D Systems) and 200 µM AAP, for inducing differentiation and maturation towards a dopaminergic neuronal phenotype. Each stage was applied for 1-week, and different combinations of stages were examined.

#### Single-Stage Dopaminergic Neuronal Differentiation (SingleDA)

The SingleDA method was performed as recently reported [32]. Briefly, hMSCs were seeded into 6-well plates (1×10^5^ cells/plate) with MSC expansion medium. After 1-day, expansion medium was replaced with Neurobasal medium (Gibco), 0.25x B-27 supplement (Gibco), 250 ng/mL SHH, 100 ng/mL FGF-8 (mouse) (R&D Systems), and 50 ng/mL FGF-2. Cells were harvested after 6- and 12-days. Media was not replaced during this period.

### Biopsy, Culture and Labeling of Human OECs

OECs were obtained frozen from the cell bank of the National Centre for Adult Stem Cell Research (Griffith University). OECs were generated from olfactory mucosa biopsies of human volunteers, in accordance with published procedures [43]. OECs were genetically labeled to express recombinant Green Fluorescent Protein (hrGFP), by transduction with pFB-hrGFP retroviral supernatant (Stratagene), according to manufacturer' instructions. OECs were FACS-sorted to isolate a population of >99% hrGFP-expressing cells, and maintained in DMEM/HAM F12 medium (Gibco) containing 10% fetal calf serum and penicillin/streptomycin. Details of the phenotype of these cells are published [53].

### RNA Extraction and Real-Time PCR

Total RNA was extracted using TRIzol reagent (Invitrogen) and RNeasy micro kit with DNase I treatment (Qiagen, Basel, Switzerland). Total RNA (1 µg) was reverse transcribed with Superscript III (Invitrogen) using 25 ng random hexamers and 1.25 µM Oligo(dT)_20_ primers, according to manufacturer' recommendations, and with RNase H treatment. Real-time RT-PCR was performed using Platinum SYBR Green qPCR SuperMix-UDG (Invitrogen) on a Rotor-Gene RG3000 machine (Corbett Research, NSW, Australia). Further details are available online in Materials and Methods S1 and Table S1.

### Stereotactic Intracerebral Transplantation

Adult male 6-OHDA unilaterally-lesioned (SN pars compacta) Wistar Ob rats (250-350 g) were obtained from the Integrative Neuroscience Facility (Howard Florey Institute, VIC, Australia). Rats were determined to have greater than 80% loss of SN pars compacta neurons by amphetamine-induced rotational testing.

Undifferentiated hMSCs (P6) and neuronal-primed hMSCs (MultiDA Stage-1) were harvested by trypsinization, washed twice, and resuspended in Hank' Balanced Salt Solution (HBSS; Sigma-Aldrich) at 3×10^4^ cells/µL and 5×10^4^ cells/µL, respectively. A total of 9×10^4^ undifferentiated hMSCs (3 µL) were injected into the lesioned striatum (*n* = 8; harvest at 2-months), and 1×10^5^ neuronal-primed hMSCs (2 µL/site) were injected into the lesioned striatum and SN (*n* = 3; harvest at 1-, 7- and 21-days). OECs were harvested similarly and resuspended in HBSS at 2.5×10^4^ cells/µL, together with neuronal-primed hMSCs (total of 5×10^4^ OECs/site).

For stereotactic transplantation, rats were anesthetized with isofluorane gas (Affane, Bomac, NSW, Australia), and placed in a stereotaxic frame with microinjector unit (Kopf Instruments, CA, USA). Cell suspensions were injected into the lesioned hemisphere with a 27-gauge needle fitted to a Hamilton syringe (Hamilton Company, NV, USA) at co-ordinates: striatum (AP:1.2, L:-2.5, V:5.0), SN (AP:-5.2, L:-3.2, V:-7.4). A cavity was created for the injection through overshooting by 200 µm. After waiting 2-min the cell suspension was injected at 1 µL/min. The needle was left in place for 2-min and slowly withdrawn. Sham-operated animals were infused with HBSS alone. All animals were immunosuppressed with daily cyclosporine A (10 mg/kg; subcutaneous; Sandimmune, Sandoz Pharmaceutical, NJ, USA; similar to [8]) from 3-days prior to grafting until harvest.

### Tissue Processing

Tissue was harvested following transcardial perfusion with phosphate buffered saline followed by Zamboni' Fixative, and processed with further fixation and embedding in polyethylene glycol. Sectioning was performed at room temperature on a rotary microtome to obtain 30 µm thick sections. See online Materials and Methods S1 for further details.

### Immunofluorescence Staining and Analysis

For details see online Materials and Methods S1. Primary antibodies included: mouse monoclonal antibodies (all IgG) against nestin (NES), microtubule-associated protein-2 (MAP-2), neuronal nuclear antigen (NeuN), TH, Glial Fibrillary Acidic Protein (GFAP)-Cy3, and Human Nuclear Antigen (HuNuc; clone 235-1); and rabbit polyclonal antibodies against Fibronectin, GFAP, β-tubulin III, and Iba-1. Alexa Fluor 488 or 594 highly-cross-adsorbed goat anti-rabbit or anti-mouse secondary antibodies were used. Negative controls consisted of secondary antibody application alone (*in vitro*). Contralateral brain regions served as internal controls (*in vivo*). Confocal imaging was performed using a Leica laser-scanning confocal microscope (DM IRE2 TCS SP2 AOBS; Leica Microsystems, Wetzlar, Germany). Individual color channels were captured separately and merged in Adobe Photoshop. Co-localization of HuNuc and DAPI signals were confirmed through analyzing z-series confocal reconstructions and corresponding orthogonal planes under 40x and 100x. Contralateral brain regions served as internal controls to confirm primary antibody specificity. Images presented are ‘aximum projection images’ from z-stacks, with 10 images captured over the thickness of the section (30–45 µm).

### Statistical Analysis

Gene expression data were expressed as mean fold-change in mRNA expression + SEM relative to control undifferentiated hMSCs. The student' *t*-test was performed to determine significant differences (*p*<0.05) using Microsoft Excel.

## Results

### *In Vitro* Dopaminergic Neuronal Differentiation of hMSCs Induces Neuronal-like Morphology

Prior to differentiation hMSCs possessed a fibroblast-like appearance (Ctrl in Fig. 1B and D), and exhibited standard MSC properties, as previously reported [28]. The three growth factor-based treatments produced cells with similar neuronal-like bipolar or multipolar morphology, with formation of refractile cell bodies and long thin processes, occasionally containing branching or minute protuberances (Fig. 1B-D). These changes occurred gradually over the differentiation period. Treatment with the MultiDA protocol yielded the most distinct morphological alterations (Fig. 1B). Following stage (S) 1, hMSCs developed a bipolar, refractile appearance and formed neurosphere-like clusters. Subsequently, in S2 and S3 many cells returned to a flatter appearance. Adding S1 growth factors to later stages (#) maintained the bipolar refractile morphology, however, uninterrupted treatment with S1 growth factors resulted in less complex branching. A considerable decrease in cell number was also observed with each stage (trypan blue cell count; data not shown). Application of the recently reported SingleDA method [32] generated a neuronal-like morphology similar to published results (Fig. 1D), which also resembled SingleND-treated cells (Fig. 1C; [21]); however, neither resulted in neurosphere-like formation. SingleND also elicited reduced cell numbers, while SingleDA yielded slight increases (data not shown).

### Increased Neural Gene Expression After *In Vitro* hMSC Neuronal Differentiation

**Figure 2:**
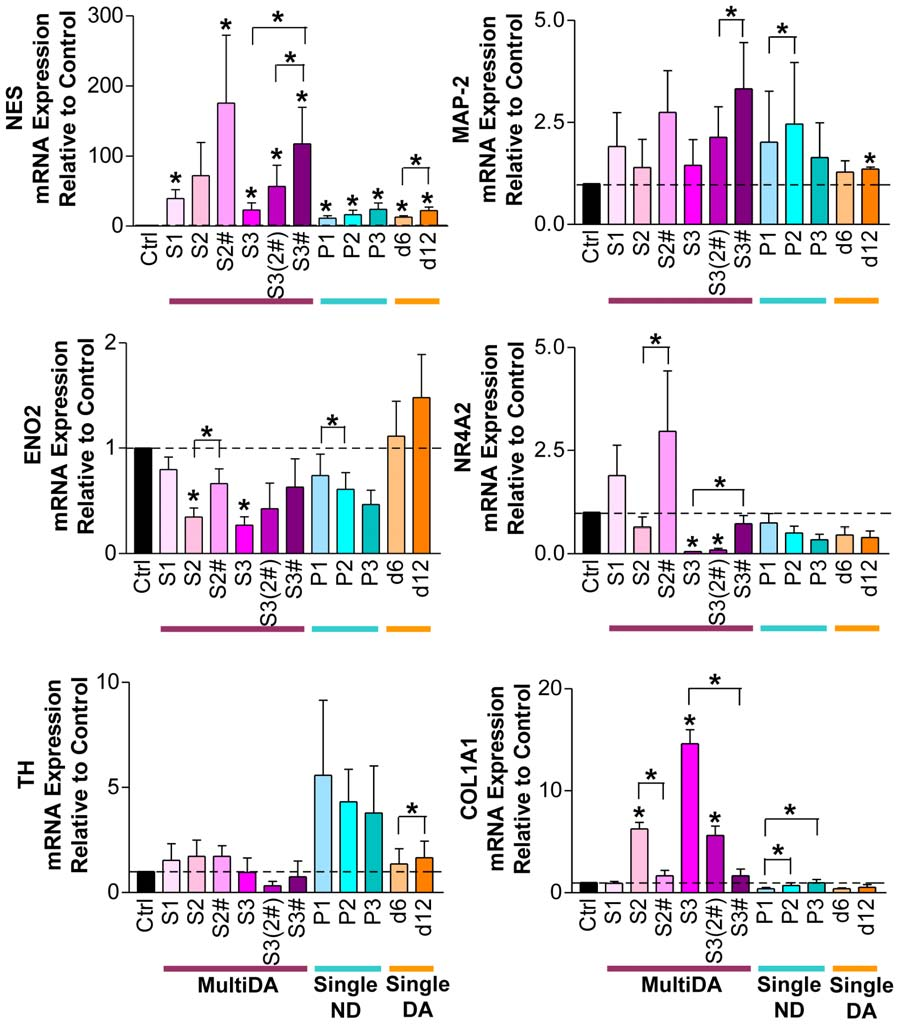
Increased neural gene expression in hMSC cultures undergoing *in vitro* neuronal and dopaminergic neuronal differentiation. Real-time RT-PCR results from the MultiDA (purple shades), SingleND (blue shades), and SingleDA (orange shades) [32] methods. Neuronal genes: *NES*, *MAP-2* and *ENO2*. Dopaminergic neuronal genes: *NR4A2* and *TH*. Mesodermal gene: *COL1A1*. Results are depicted as mean fold change in mRNA expression + SEM relative to control undifferentiated hMSC cultures, with baseline set at 1.0 (*n* = 3). * *p*<0.05 compared with the control, unless indicated by brackets. Ctrl, control undifferentiated hMSCs; S, stage; #, growth factors from S1 included; P, passage; d, day.

**Figure 3:**
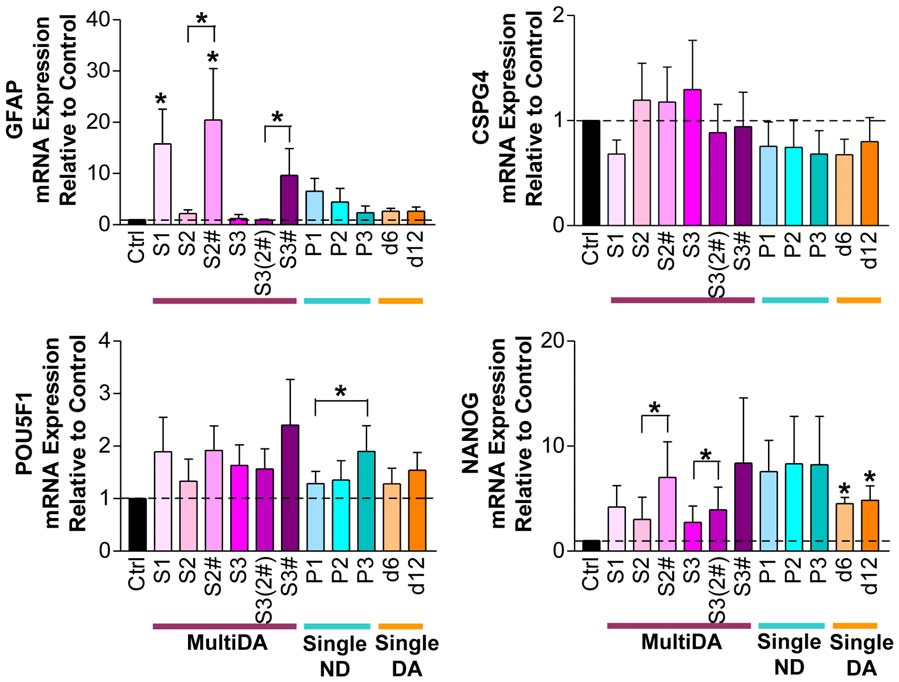
Increased neural gene expression in hMSC cultures undergoing *in vitro* neuronal and dopaminergic neuronal differentiation. Real-time RT-PCR results from the MultiDA (purple shades), SingleND (blue shades), and SingleDA (orange shades) [32] methods. Glial genes: *GFAP* and *CSPG4*. Pluripotency genes: *POU5F1* and *NANOG*. Results are depicted as mean fold change in mRNA expression + SEM relative to control undifferentiated hMSC cultures, with baseline set at 1.0 (*n* = 3). * *p*<0.05 compared with the control, unless indicated by brackets. Ctrl, control undifferentiated hMSCs; S, stage; #, growth factors from S1 included; P, passage; d, day.

Expression of neuronal, dopaminergic neuronal, glial, mesodermal, and pluripotency genes were examined (Fig. 2 and Fig. 3). Neuronal progenitor marker *NES*, and neuronal markers, *MAP-2* and neuron-specific enolase (*ENO2*), were detected in all samples before and after differentiation. *NES* was significantly up-regulated in all conditions, apart from MultiDA S2. The MultiDA procedure induced the highest levels of *NES*, and addition of S1 growth factors appeared beneficial for *NES* expression. Human MSCs also expressed nuclear receptor subfamily 4, group A, member 2 (*NR4A2*; also *Nurr1*; transcription factor essential for midbrain dopaminergic differentiation) and *TH* (rate-limiting enzyme in catecholamine synthesis) prior to and following induction. A trend of increasing *NR4A2* expression could be observed in MultiDA S1 and S2#. Both single-step methods resulted in decreasing *NR4A2* expression. The highest levels of *TH* expression were observed with SingleND. Glial gene *GFAP* was significantly up-regulated in MultiDA S1 and S2#, but was not significantly changed in all other conditions. Expected down-regulation of mesodermal marker collagen I (*COL1A1*) was not observed with any method, and instead, *COL1A1* was significantly increased in MultiDA without FGF-2/EGF. Only weak expression of pluripotency markers POU class 5 homeobox 1 (*POU5F1*; also *OCT3/4*) and Nanog homeobox (*NANOG*) were detected.

### Increased Expression of Early Neuronal Proteins After *In Vitro* hMSC Neuronal Differentiation

**Figure 4:**
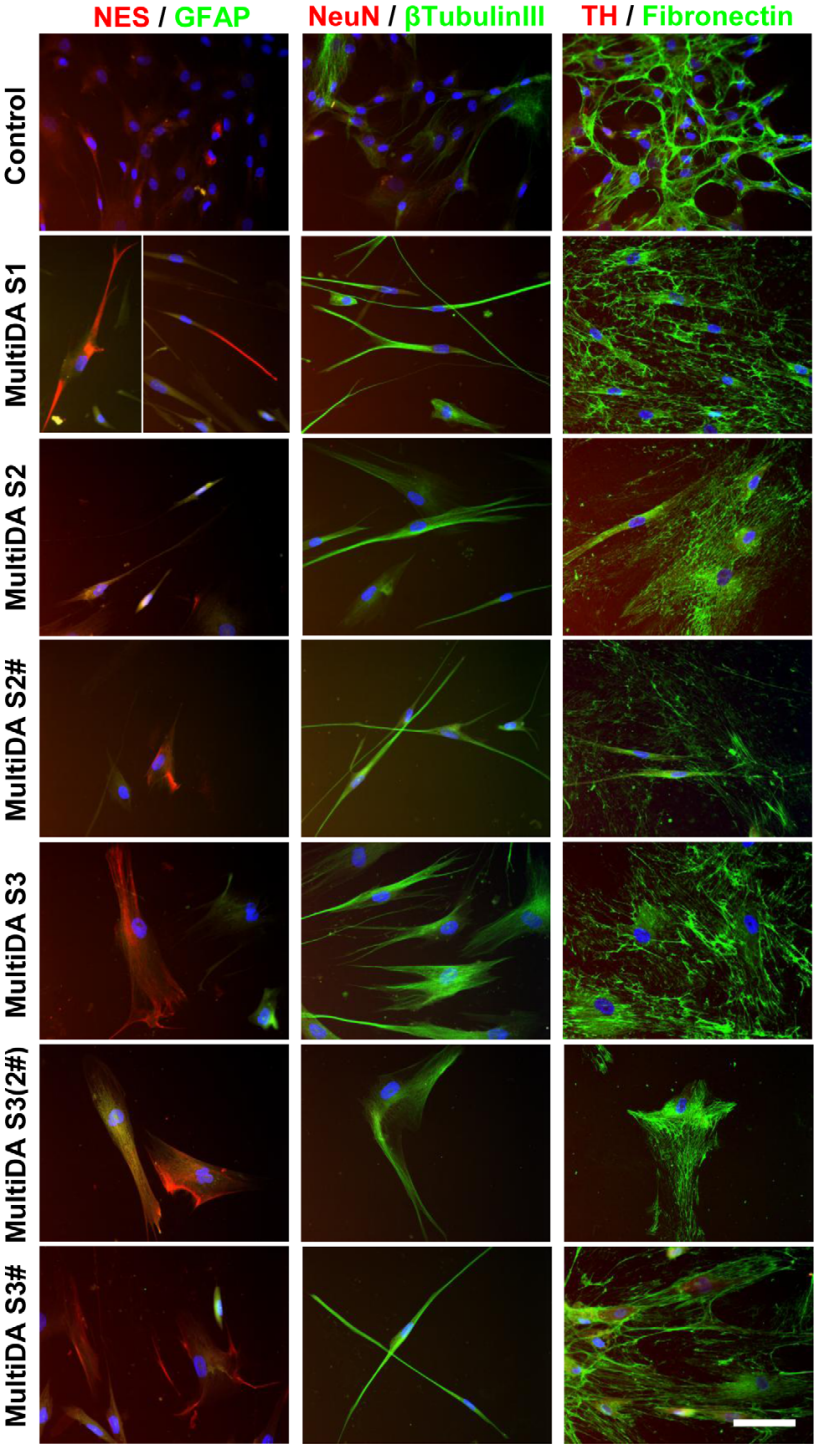
Increased early neuronal protein expression by hMSCs during *in vitro* neuronal and dopaminergic neuronal differentiation. Representative images depicting immunofluorescence staining of control undifferentiated hMSC cultures and cultures treated with the MultiDA procedure (*n* = 3). Cells were examined for expression of neuronal progenitor marker NES, astroglial marker GFAP, neuronal markers NeuN and β-tubulin III, dopaminergic neuronal marker TH, and mesodermal marker fibronectin. Growth factor-treated cells were observed to express NES, and up-regulated β-tubulin III expression, but other neural markers were not detected. Cells also continued to strongly express fibronectin. These results suggest that the MultiDA method was only capable of generating immature neuronal-like cells. Nuclei were counterstained with DAPI. Negative controls consisted of secondary antibody application alone (not shown). S, stage; #, growth factors from S1 included. Scale bar: 50 µm.

**Figure 5:**
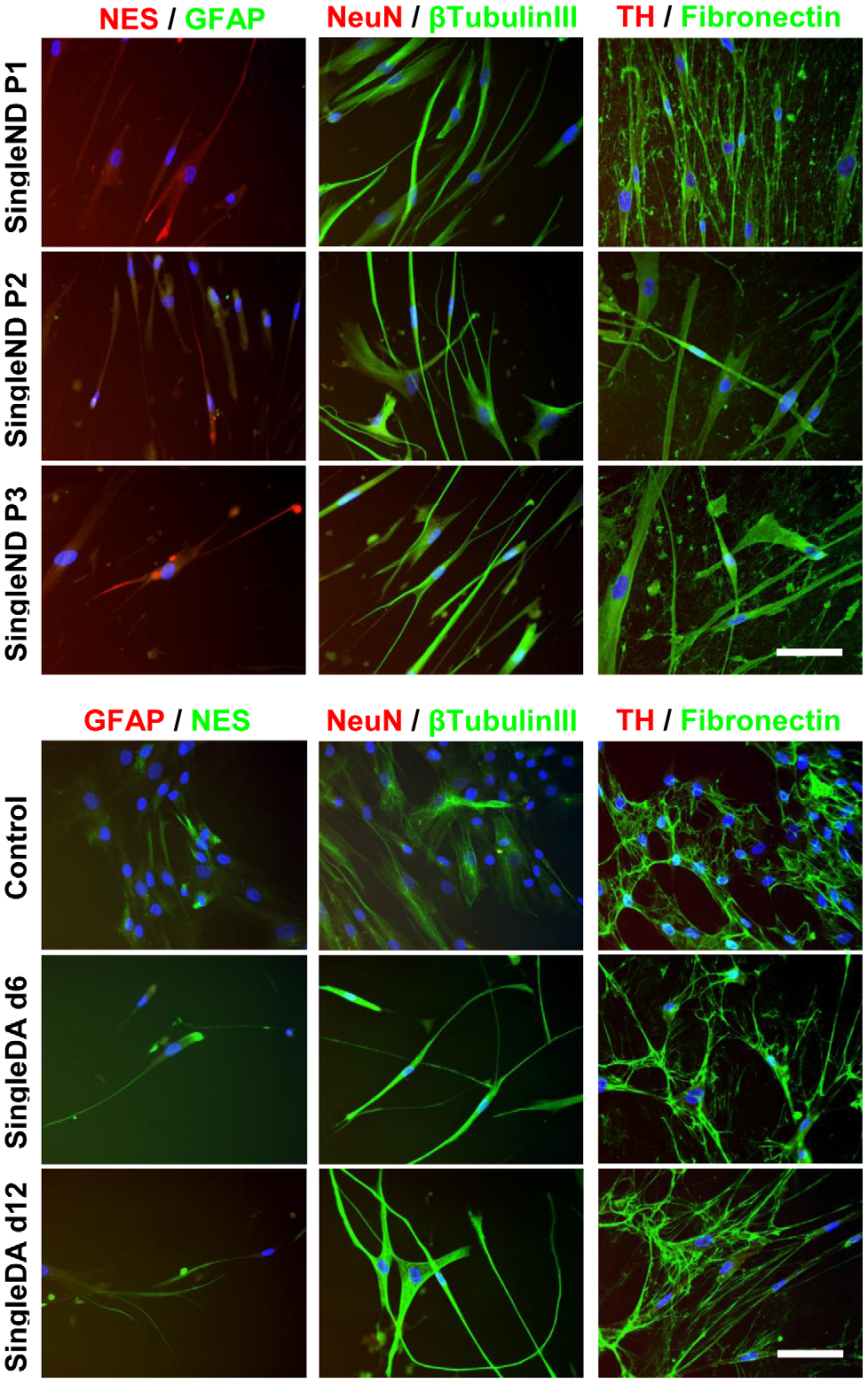
Increased early neuronal protein expression by hMSCs during *in vitro* neuronal and dopaminergic neuronal differentiation. Representative images depicting immunofluorescence staining of control undifferentiated hMSC cultures and cultures treated with the SingleND and SingleDA procedures (*n* = 3). Cells were examined for expression of neuronal progenitor marker NES, astroglial marker GFAP, neuronal markers NeuN and β-tubulin III, dopaminergic neuronal marker TH, and mesodermal marker fibronectin. Growth factor-treated cells were observed to express NES, and up-regulated β-tubulin III expression, but other neural markers were not detected. Cells also continued to strongly express fibronectin. These results suggest that all 3 neuronal differentiation methods investigated were only capable of generating immature neuronal-like cells. Nuclei were counterstained with DAPI. Negative controls consisted of secondary antibody application alone (not shown). P, passage; d, day. Scale bar: 50 µm.

PCR findings were confirmed by immunofluorescence staining for protein expression (Fig. 4 and Fig. 5). Consistent with PCR, NES was detected in both undifferentiated control hMSCs (<10% positive) and at all time points examined during neuronal differentiation (<20% positive). NES protein occurred in a cytoskeletal pattern, and appeared slightly up-regulated following all differentiation methods examined. The percentage of positively-stained cells may be underestimated due to the loss of neuronal-like cells during slide processing, since these cells were loosely attached to culture surfaces. Prior to differentiation, some hMSCs expressed low levels of β-tubulin III (<15%). Following differentiation, >80% of cells exhibited cytoskeletal β-tubulin III staining, except in SingleND (>90% positive) and MultiDA S2 and S3 without FGF-2/EGF (60–80%) conditions. As expected, fibronectin was strongly expressed by undifferentiated hMSCs (90–100%) in an extracellular fibrous pattern. Continued fibronectin expression was detected following differentiation (>90%), except in MultiDA S3, which showed slightly lower proportions (>80%) of fibronectin expression and weaker staining intensity. Other markers examined (GFAP, NeuN and TH) were absent from the majority of cells and weak expression could only be detected occasionally (not shown).

### Limited Survival of hMSCs and OECs Post-Transplantation

**Figure 6:**
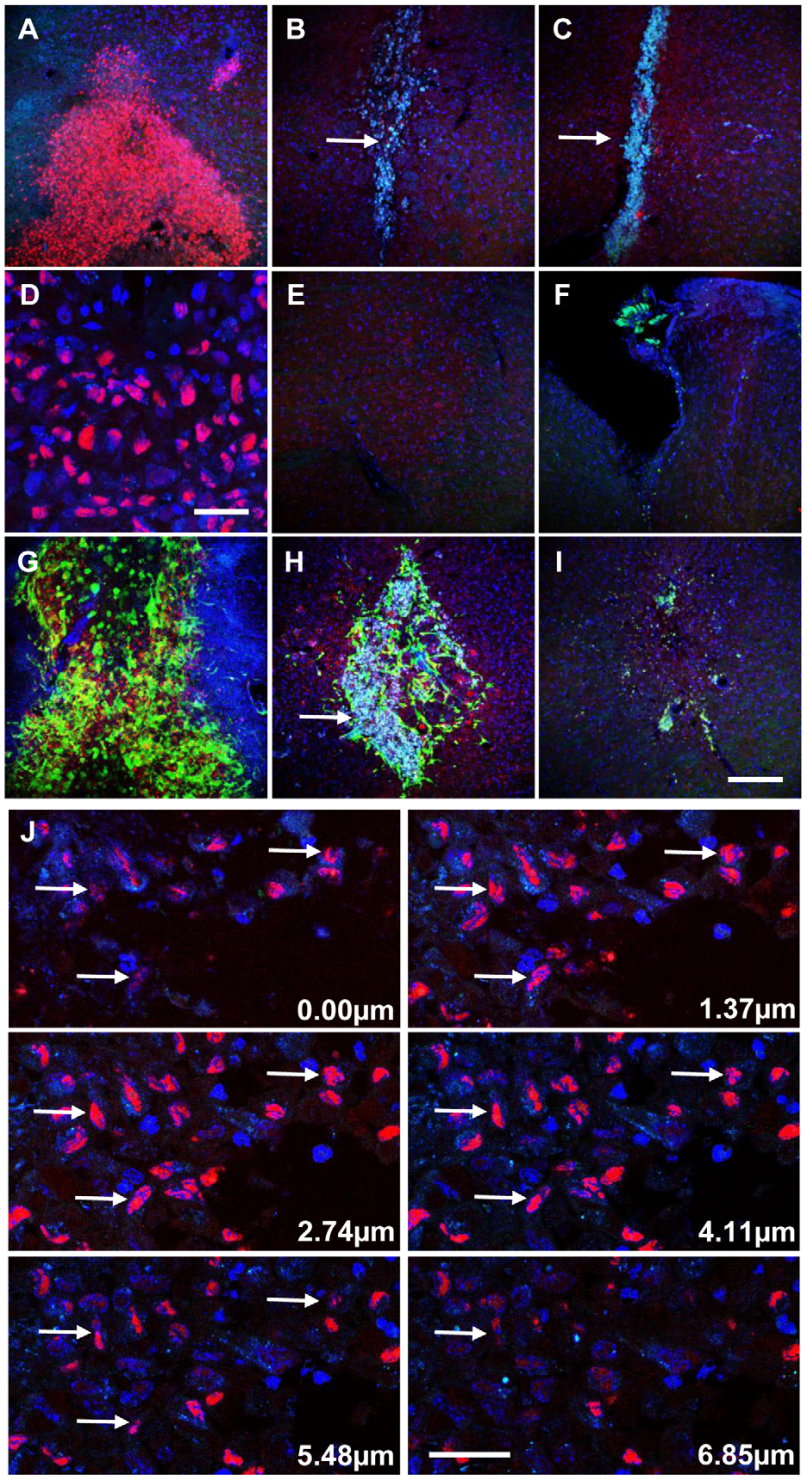
Evidence of limited survival of neuronal-primed hMSCs in the lesioned hemisphere of the hemiparkinsonian rat. The survival and engraftment of neuronal-primed hMSCs and GFP-positive OECs (green) were examined by immunohistological staining using an antibody against Human Nuclear Antigen (HuNuc; red). Representative images following transplantation of (A–F) neuronal-primed hMSCs alone, and (G-I) co-transplanted with OECs (*n* = 3; maximum projection z-stacks shown (35 µm)). Surviving human cells could be detected at 1-day post-transplantation (A and G). However, by 7-days little HuNuc signal remained (B and H), and this could no longer be detected by 21-days (C and I). Higher magnification view of (A) is shown in (D), with HuNuc staining seen to be co-localised with DAPI-stained nuclei (blue). Increasing presence of autofluorescent matter (aqua) within the graft core was observed in parallel with the loss of human cells (arrows in B, C, and H). This autofluorescence and HuNuc staining were not observed in the corresponding regions in the contralateral hemisphere (representative image shown in (E)). The majority of human cells were concentrated in the needle tract with few, if any, detected at the needle entry site (F). Similar findings were observed in both striatal and nigral graft sites. (J) Confocal z-series identification of human cells. Examination of z-series images from graft sites confirms co-localisation of HuNuc (red) signal with DAPI-stained nuclei (blue) within the nigral graft site at 1-day (arrows). Similar results were obtained for striatal graft sites and OEC co-transplanted animals, as well as at 7-days. Scale bar: 150 µm in (I) for (A–C, E–I); 30 µm for (D) and (J).

To identify transplanted human cells, graft regions were examined by HuNuc staining, and GFP fluorescence for OEC identification (Fig. 6). Cells displaying co-localization of HuNuc and DAPI were found within striatal and nigral graft sites at 1-day post-transplantation (Fig. 6A and G). Surviving hMSCs were visible as a mass of cells toward the end of the needle tract, with few at the needle entry point (Fig. 6F), and none in the contralateral hemisphere (Fig. 6E). Over time HuNuc staining decreased, with little signal remaining at 7-days (Fig. 6B and H), and complete absence by 21-days (Fig. 6C and I). In addition, autofluorescent cell debris and lipofuscin-like material were found within graft cores, which increased in parallel with HuNuc loss, and reduced graft size and cellular density. No difference was observed between grafts containing neuronal-primed hMSCs alone or in combination with OECs. Concomitant loss of the OEC-derived GFP signal provides further support for the transient *in vivo* survival of human cells. Analysis of undifferentiated hMSC grafts at 2-months post-transplantation also revealed absence of engraftment, with graft cores also containing autofluorescent debris (results not shown). Confocal z-series analysis showed co-localization of HuNuc signals with DAPI-stained nuclei (arrows) for both hMSC only and OEC co-transplanted grafts at 1-day post-transplantation (Fig. 6J), excluding possible misinterpretation due to overlaying fluorescent signals.

### Accumulation of Astrocytes and Microglia/Macrophages at Graft Sites

**Figure 7:**
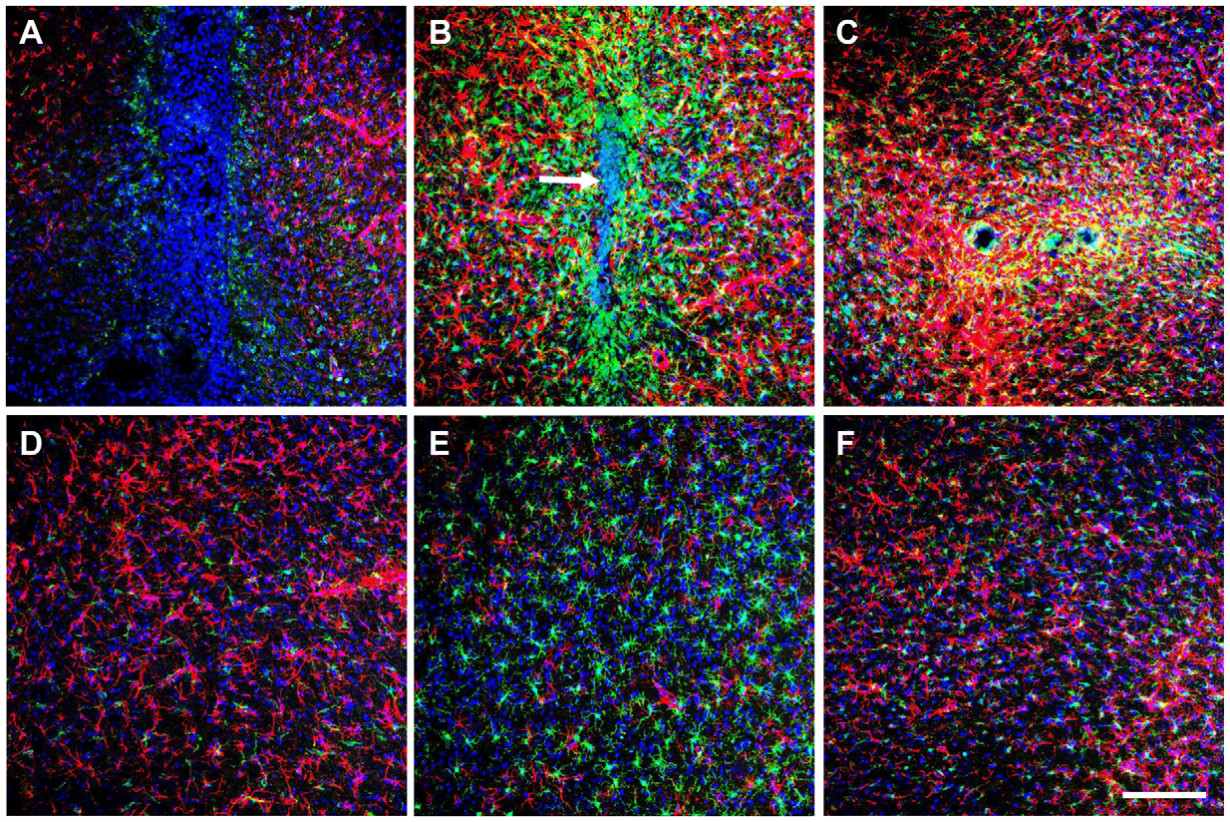
Increasing glial accumulation at graft sites during the initial 3-weeks post-intracerebral transplantation in hemiparkinsonian rats. Immunohistological analysis revealed the accumulation of GFAP-positive astrocytes (red) and Iba-1-positive microglia/macrophages (green) at graft sites (*n* = 3). Representative images depicting (A–C) neuronal-primed hMSC graft sites in the striatum, and (D–F) corresponding region controls in the contralateral hemisphere, at 1-day (left column), 7-days (middle column) and 21-days (right column). (A) Initially, at 1-day post-transplantation little GFAP or Iba-1 was detected. (B) By 7-days grafts were densely surrounded by a layer of GFAP-positive and Iba-1-positive cells, and contained an autofluorescent core (white arrow). (C) At the end of the 21-day period a marked astrogliosis was present in the graft region. Both striatal and nigral grafts exhibited this glial accumulation, which suggests the presence of an inflammatory response at graft sites. (D–F) This pattern of glial distribution was absent from the contralateral hemisphere. Images shown are maximum projection z-stacks. Nuclei have been counterstained with DAPI (blue). Scale bar: 150 µm.

To investigate the loss of hMSCs and OECs, graft sites were characterized for the presence of glia. Striatal and nigral grafts displayed massive infiltration by Iba-1-positive microglia/macrophages and GFAP-positive astrocytes, which increased over the 3-week period (Fig. 7A–C). By 7-days, microglia/macrophages were present in a radial pattern around the graft core. Increased astrocytic presence was also found surrounding the graft site and extending beyond the microglia/macrophage layer. Undifferentiated hMSC grafts examined at 2-months also showed evidence of astrocytic infiltration and/or proliferation at graft sites and along needle tracts (data not shown). Furthermore, these microglia/macrophages displayed an activated appearance, with intense Iba-1 staining [54], and altered morphology including retraction and thickening of processes and increased cytoplasmic area [54], [55].

Corresponding regions in the contralateral hemisphere did not exhibit glial accumulation (Fig. 7D-F), and microglia possessed quiescent morphology (small cell bodies and fine cytoplasmic ramifications). Interestingly, sham controls showed some evidence of glial accumulation, suggesting that this may be a response to injury caused by needle insertion (data not shown).

### Absence of Endogenous Host Neuronal Differentiation Post-Transplantation

**Figure 8:**
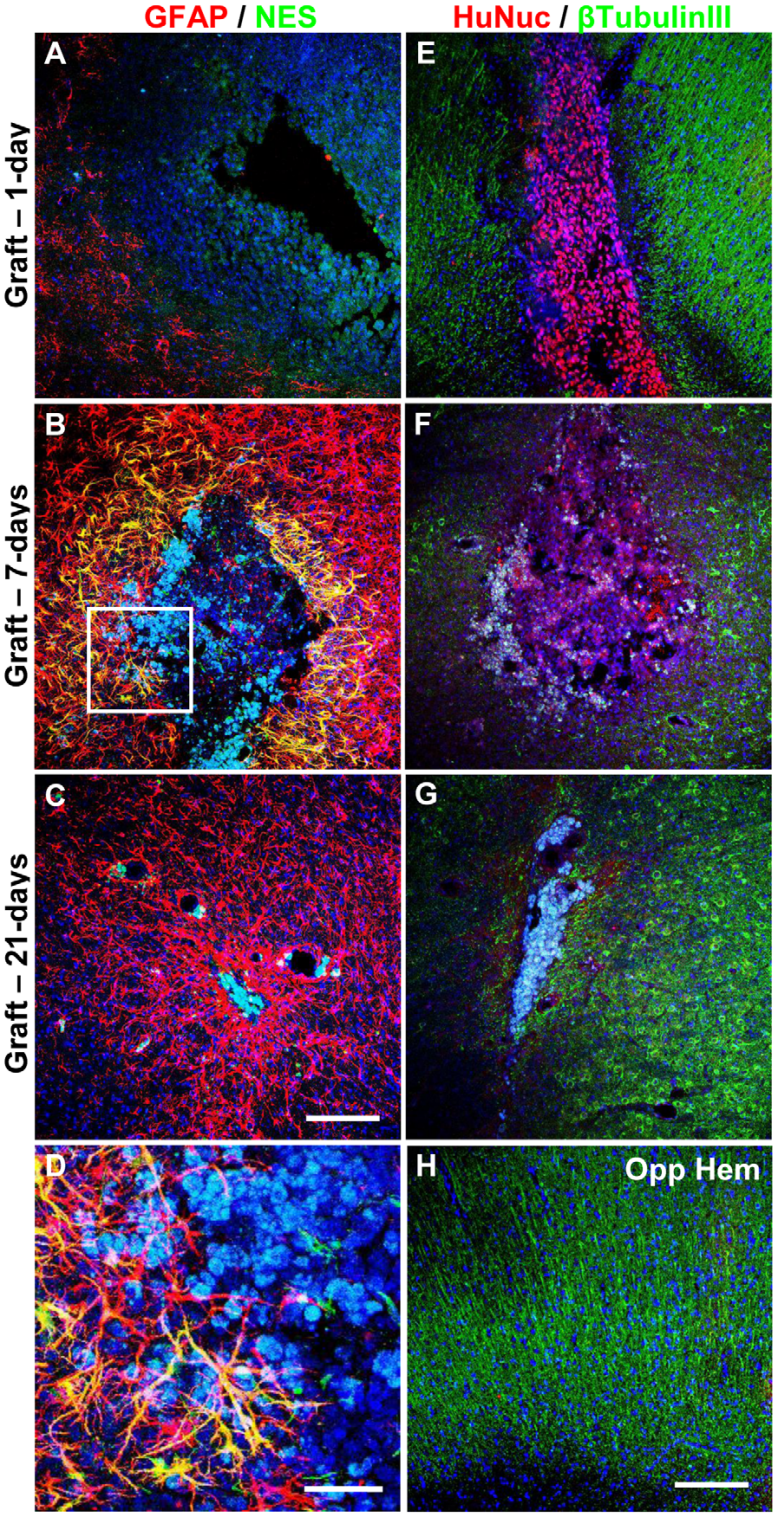
Absence of endogenous host neuronal differentiation and neuronal marker expression within neuronal-primed hMSC graft sites. Striatal and nigral grafts were examined for the presence of NES (green) and GFAP (red) at (A) 1-day, (B) 7-days, and (C) 21-days post-transplantation (*n* = 3). (D) Higher magnification view of boxed area in (B). Rare NES-positive cells were only detected within the graft core or in the immediate astroglial boundary at 7-days. NES-positive activated astrocytes could also be detected. Graft sites were also examined for the presence of β-tubulin III (green) and human nuclear antigen (HuNuc; red) at (E) 1-day, (F) 7-days, and (G) 21-days post-transplantation (*n* = 3). Expression of β-tubulin III was missing from the region containing the graft sites, and was primarily found surrounding the graft core; while, the corresponding regions in the contralateral hemisphere (Opp Hem) exhibited evenly distributed β-tubulin III expression (representative image shown in (H), which is contralateral to (E)). Similar results were observed in OEC co-transplanted recipients. Representative images shown are maximum projection z-stacks (40 µm). Nuclei were counterstained with DAPI (blue). Scale bar: 150 µm in (C) for (A–C); 40 µm for (D); 150 µm in (H) for (E–H).

Graft regions were examined for host neuronal progenitor activity because hMSC transplantation has been proposed to elicit host responses that may account for functional improvement. Immunohistological analysis for NES (Fig. 8A–D) revealed little, if any, NES immunoreactivity at any period. Host cells that expressed NES were predominantly GFAP-positive astrocytes immediately surrounding grafted cells, indicating activation [56]. A few rare NES-positive cells could be detected within the graft core and at astrocytic borders at 7-days (Fig. 8D). The location of the NES-positive cells suggests that this staining is unlikely to represent host neuronal progenitor involvement. Instead, NES expression may originate from neuronal-primed hMSCs that express NES gene and protein, and is consistent with absence of NES at 21-days. No differences were observed between striatal and nigral grafts of neuronal-primed hMSCs alone or with OECs.

### Lack of Neuronal Marker Expression by Transplanted hMSCs at Graft Sites

Since NES was detected in a small proportion of cells within the graft, further characterization for neuronal markers was conducted. Neuronal β-tubulin III immunoreactivity was not observed in the transplantation region (Fig. 8E–G), whereas the contralateral region showed consistent evenly distributed expression (Fig. 8H), suggesting that surgical trauma caused loss of host β-tubulin III. Similar results were observed with OEC co-transplantation.

**Figure 9:**
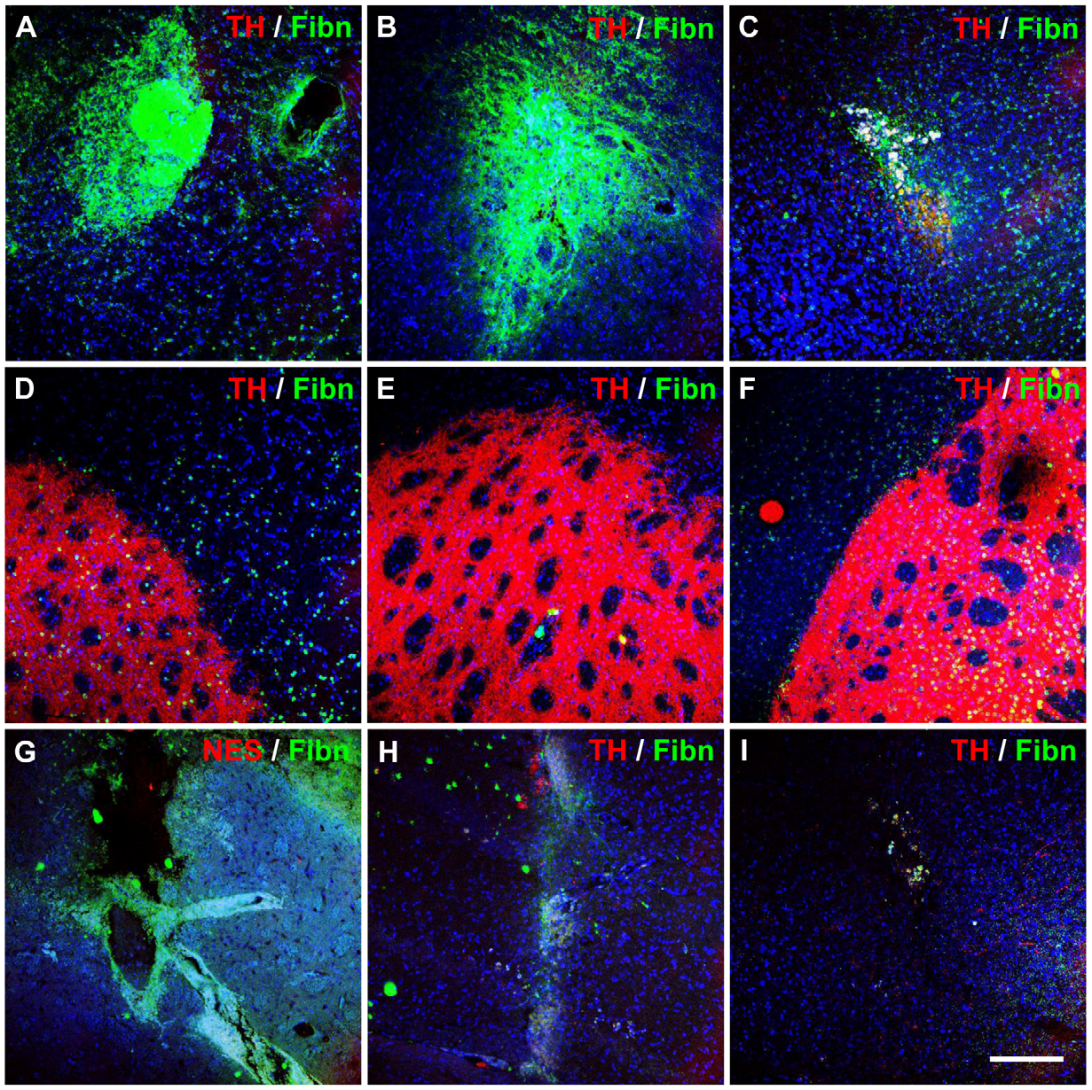
Matrix deposition and lack of dopaminergic neuronal differentiation after transplanting neuronal-primed hMSCs and OECs. Fibronectin and TH expression were assessed in striatal and nigral grafts at 1-day (left column), 7-days (middle column) and 21-days (right column). Representative images are shown of (A-C) neuronal-primed hMSC graft sites in the striatum, (D–F) corresponding regions in the contralateral hemisphere, and (G–I) injection sites in sham controls (*n* = 3). Immunohistological analysis revealed a fibronectin-positive matrix surrounding hMSC graft sites from 1-day post-transplantation (A and B), however, this was markedly reduced by 21-days (C). The concomitant loss of transplanted cells suggests that the fibronectin matrix was deposited by the graft. Furthermore, sham injected animals did not exhibit dense fibronectin expression (G–I). TH expression was not detected in any graft sites or sham-injected sites, and was only expressed in the unlesioned hemisphere (D–F). Similar results were observed in OEC co-transplanted recipients. Images shown are maximum projection z-stacks. Nuclei were counterstained with DAPI (blue). Scale bar: 150 µm.

Staining for TH confirmed strong unilateral expression within varicose nerve fibres in the unlesioned hemisphere (Fig. 9D–F), with a complete absence of TH-localizing nerve fibres within the striatum of the lesioned hemisphere. TH staining was predominantly absent from graft sites (Fig. 9A–C), regardless of the transplantation site or whether OECs were co-transplanted. Sham-treated animals also did not exhibit TH expression at injection sites (Fig. 9H and I).

### Extracellular Matrix Deposition at Graft Sites

A dense fibronectin-positive matrix was observed surrounding neuronal-primed hMSC grafts as early as 1-day post-transplantation, and decreased over time with little remaining at 21-days (Fig. 9A–C). Similar findings were obtained with co-transplantation of OECs at both striatal and nigral sites (not shown); while only slight fibronectin expression was detected contralaterally (Fig. 9D–F). Sham controls displayed very little, if any, fibronectin deposition (Fig. 9G–I). This suggests that the dense fibronectin matrix was produced by the transplanted human cells, rather than being deposited by host cells during scar formation. Fibronectin decreased considerably in parallel with hMSC and OEC loss, supporting this explanation. Graft-derived fibronectin production is also supported by continued expression of fibronectin protein by neuronal-primed hMSCs *in vitro* (Fig. 4 and Fig. 5).

## Discussion

In this report we have shown that: 1) treatment of hMSCs with three growth factor-based neuronal differentiation procedures elicited similar immature neuronal-like phenotypes, with continual FGF-2/EGF supplementation achieving neurosphere-like formation and the highest expression of *NES* and *NR4A2*; 2) hMSCs survived transiently in the lesioned hemisphere of immunosuppressed Parkinsonian rats; 3) activated astrocytes and microglia/macrophages accumulated around graft sites, indicating the presence of an inflammatory response; 4) further differentiation of neuronal-primed hMSCs was not observed *in vivo*, and neuronal-primed hMSCs continued producing fibronectin matrix; 5) hMSC-derived neurotrophic effects on host neuronal progenitors/cells were not apparent in complete-lesion Parkinsonian rodents; and 6) co-transplantation of OECs did not enhance graft survival or differentiation.

Previously, we showed that application of growth factors and neural-inducing culture conditions resulted in immature neuronal-like differentiation of hMSCs [21], [28]. In the present study, we endeavored to induce a dopaminergic phenotype through sequential application of growth factors important in midbrain dopaminergic neuronal development. With a range of methods reported and no consensus on an optimal method, we also conducted a comparison of published methods. Despite the different growth factors, surface coatings and culturing procedures employed, all three methods examined only generated immature neuronal-like cells. Nevertheless, MultiDA S1 yielded the greatest increases in *NES* and *NR4A2* expression, as well as neurosphere-like formation and enhanced cell survival compared to S2 and S3, and as a result was selected for *in vivo* experiments. Observations that cultures were not maintained well in later stages of the MultiDA system raises questions regarding the responsiveness of hMSCs to SHH, FGF-8 and GDNF. Previous reports showed these growth factors to be beneficial for *in vitro* MSC dopaminergic neuronal differentiation [22], [32], [33], [35]. In addition, MSCs are known to functionally express both components of the SHH receptor, as well as FGF receptors *FGFR1* and *FGFR4* [32], [57]; however, only part of the GDNF receptor was expressed, rendering MSCs incapable of responding to GDNF. This suggests variability in MSC expression of growth factor receptors, and may account for the inability of MultiDA S3 to elicit the expected differentiation effects.

The stage of SC differentiation that is optimal for transplantation purposes has not been well defined, although it is believed that immature neuronal cells possess the greatest potential for successful engraftment and integration into host circuitry [20], [23]. The growth factor-based methods employed here were successful in driving hMSCs towards an early neuronal fate; however, cells continued expressing mesodermal and pluripotency markers, suggesting that further neuronal differentiation may be required. Future experiments are warranted for investigating whether the electrophysiological properties of these neuronal-like cells resemble that of immature neurons. The minimal expression of TH protein suggests that dopamine is not produced, and additional agents or cell-to-cell contact with neural support cells may be required for generating dopamine-secreting cells. Another comparative study also found continued expression of mesodermal marker fibronectin after application of different neuronal differentiation methods [58]. These findings highlight the importance of showing concomitant down-regulation of mesodermal and MSC markers with neuronal differentiation, which has not been commonly examined. MSC neuronal differentiation could be improved by addition of fatty acids, such as docosahexaenoic acid and arachidonic acid [59], inflammatory mediator IL-1α [29], [60], and retinoic acid, Brain-Derived Neurotrophic Factor and/or Nerve Growth Factor [32].

Transplantation of undifferentiated and neuronal-primed hMSCs into the lesioned hemisphere of immunosuppressed hemiparkinsonian rats yielded transient graft survival. A number of factors may be responsible for this, including: the presence of innate inflammatory responses that may have been initiated by surgical trauma during transplantation; the xenogeneic nature of the graft; and the ‘foreign’ nature of hMSCs, which had not gained a mature neuronal phenotype as evidenced by continued fibronectin production. It is also possible that the lesioned adult rat striatum and SN may lack the appropriate factors for supporting hMSC survival, engraftment and differentiation. These findings contradict earlier reports [33], [35], [36], [61]–[64], which have demonstrated some level of MSC-induced repair in Parkinsonian models; however, shows similarity with recent reports describing limited MSC survival post-transplantation [23], [65], [66]. Initial reports employed BrdU and other thymidine analogs for cell-labelling, which have since been found to result in the transfer of donor labels to host cells [65], [67]. These cell-labelling techniques may provide misleading indication of donor cell survival and differentiation. In the present study, we avoided the use of these labels and identified human cells by immunohistological detection through human-specific antibodies and GFP-transgene fluorescence (OECs). Our findings of limited MSC survival are consistent with these recent reports, and suggest that discrepancies with earlier studies may be related to the varying methods of graft detection.

The immunosuppressive effects of MSCs have primarily been demonstrated in *in vitro* studies, and initially focused on T lymphocyte effects [68]–[70]; however, the relevance to *in vivo* or clinical settings remains unclear, although the limited data available at present provides little evidence that donor MSCs are able to engraft after infusion or transplantation [71], [72]. Nevertheless, studies have reported the transplantation of allogeneic or xenogeneic MSCs into the brain of immunocompetent animals without detection of a host immune response [73]–[75]. We detected the presence of microglial/macrophage accumulation after hMSC transplantation into the lesioned hemiparkinsonian rat brain. This inflammatory response occurred in the presence of cyclosporine immunosuppression and was also present in sham controls, indicating that while it was not specific to the transplanted cells, the inflammatory response was not abolished by hMSCs. Increasing evidence that MSCs may not be intrinsically immune-privileged has been reported. An immediate inflammatory response was elicited following allogeneic MSC grafting in intact adult rat hippocampus or striatum [65]. In addition, robust cellular immune responses were generated following injection of MSCs into the striatum of allogeneic hemiparkinsonian rats [66]. Cyclosporine treatment acts primarily to inhibit T lymphocyte-mediated immunity [76], indicating the involvement of other arms of the immune system in MSC loss. Other studies have also shown that cyclosporine treatment is suboptimal for xenotransplantation of neural cells into neural regions [77], and that complement factors, immunoglobulins and macrophages are involved in responses against discordant xenografts [78]–[80]. Whilst, transplantation of MSCs has been performed without immunosuppression [35], [73]–[75], or with cyclosporine-based [23], [61], [64], [81] or FK506-based [33] immunosuppression, our findings suggest that suppression of the innate immune response may be required for improved graft survival. Recently, enhanced microglial suppression was employed for transplantation into degenerated retina through application of oral cyclosporine A, azathioprine, prednisolone, and indomethacin, which decreased microglial accumulation and increased SC survival [82].

Following transplantation of hMSCs alone or with OECs, we were unable to detect further signs of neuronal or dopaminergic neuronal differentiation and maturation. In line with this and the lack of engraftment, assessment of behavioural function in hemiparkinsonian rats by amphetamine-induced rotational asymmetry showed no improvement at 1 month post-transplantation of hMSCs (not shown). Coyne and colleagues also failed to observe maturation of undifferentiated MSCs following intracerebral transplantation in normal adult rats [65]. They attributed this to the limited capacity of the intact adult brain for directing the differentiation of transplanted SCs. We grafted hMSCs into the striatum, as this is the region requiring dopamine provision, as well as the SN, since the cell bodies of midbrain dopaminergic neurons are contained in the SN and specific cues for dopaminergic differentiation may be present here. However, only transient graft survival was observed in both regions in the absence of differentiation. Furthermore, fibronectin deposition was only observed at hMSC graft sites, suggesting that the hMSCs were the source of fibronectin, and therefore still possessed primitive properties. These observations are in accordance with recent work describing the transplantation of undifferentiated MSCs and MSC-derived committed neural precursor cells in a rat model of focal cerebral ischemia, which demonstrated enhanced engraftment and functional improvement with the more neurally-committed cells [83].

The mechanism by which MSCs exert functional improvements *in vivo* has been the subject of recent controversy. Pioneering studies suggested that tissue repair of different organ systems occurred via the broad developmental plasticity and transdifferentiation capacity of MSCs; our findings contribute to emerging evidence that suggests MSCs exhibit low or transient levels of *in vivo* engraftment and transdifferentiation, and may possess a more prominent role in secreting factors that alter host microenvironments [84]. Tissue repair may then occur through stimulation of survival and proliferation of host cells, modulation of immune responses, induction of angiogenesis, and reduction of apoptosis. In the present study, the absence of a host regenerative response may be related to the severe, complete-lesion hemiparkinsonian model employed where there is no chance for rescuing damaged neurons, since the process of neuronal death is complete. The high-severity lesion used here may also explain why strategies of OEC co-transplantation and nigral and striatal grafting could not improve hMSC survival, differentiation and induction of host regeneration. Recently, other studies have reported that co-grafting of OECs with olfactory nerve fibroblasts and neural SC-derived dopaminergic neurons is beneficial for dopaminergic axon transection [85] and Parkinsonian rodents [86], respectively. A supportive role for OECs was further suggested by findings that OEC transplantation alone was insufficient for promoting tissue repair and recovery in hemiparkinsonian rats [87]. Alternatively, an MSC-induced protection-restoration effect on host neural cells may be possible in a partial-lesion hemiparkinsonian model containing residual surviving dopamine terminals. Promising results of behavioral improvement have recently been obtained with this type of model and transplantation of MSC-derived neurotrophic factor-secreting cells [64].

Despite the issues raised above, the current state of research suggests that MSC-based cellular therapies are possible for neurological diseases and disorders, although are much more complicated than initially believed with involvement of distinct underlying mechanisms. Our findings highlight the need for further careful studies to resolve the controversies and inconsistencies within the field, and to allow the proper development of reproducible and reliable methods for MSC application in cellular therapies for neurological diseases. Key areas warranting further investigation include the ‘supportive’ (neurotrophic) qualities of MSCs and MSC-derived cells, and the effects of transplanting these cells in partial- versus complete-lesion Parkinsonian models.

## Supporting Information

Materials and Methods S1 Supplementary information for real-time RT-PCR, tissue processing, and immunofluorescence staining and analysis.
(DOC)

Table S1 Primer pairs for real-time RT-PCR.
(DOC)
