# Supplementary material for: Transplantation of Neuronal-Primed Human Bone Marrow Mesenchymal Stem Cells in Hemiparkinsonian Rodents
Source: PLoS One. 2011 May 23;6(5):e19025. doi: 10.1371/journal.pone.0019025 (PMC3100305; doi:10.1371/journal.pone.0019025)
Supplement: Materials and Methods S1 — Supplementary information for real-time RT-PCR, tissue processing, and immunofluorescence staining and analysis. (DOC) [file pone.0019025.s001.doc]

**Materials and Methods S1**

## Real-Time PCR

All real-time PCR reactions were performed in duplicate within the same PCR plate. The thermal profile for all reactions was: 2 min at 50°C, 2 min at 95°C, 40 cycles of 30 seconds (s) at 95°C, 30 s at 60°C and 30 s at 73°C. Melt curve analysis was also performed. The ratio of the target gene expression in experimental/control (‘fold change in target gene’/‘fold change in reference gene’) was determined using the ΔΔCt method (75). The reference genes used were hypoxanthine phosphoribosyltransferase 1 (*HPRT1*) and glyceraldehyde-3-phosphate dehydrogenase (*GAPDH*). Water amplification controls were included in each reaction to control for contaminating cDNA and genomic DNA. To exclude genomic DNA contamination, RNA was treated with RNase-free DNase I (Qiagen) and primers were intron-spanning. Primers are listed in Supplementary Table 1.

## Tissue Processing

For harvesting, subjects were anesthetized with Lethabarb-Sodium Pentabarbitone (1.5 mL/animal; Virbac Animal Health, TX, USA) and perfused transcardially with phosphate buffered saline (PBS) followed by Zambonie’s Fixative. Brains were harvested and immersed in Zambonie’s Fixative overnight under vacuum, rinsed twice in PBS (30 min), and placed in PBS-azide (0.1%). Specimens were dehydrated through graded alcohols (50% ethanol for 30 min, 70% ethanol for 30 min, 100% ethanol for 60 min), permeabilized with DMSO for 60 min, rinsed in 100% ethanol for 5 min, and placed in fresh 100% ethanol for 60 min. Brains were embedded in polyethylene glycol (PEG; Sigma-Aldrich) by placing in PEG 400 overnight at room temperature under vacuum, PEG 1000 at 48ºC under vacuum until sunk (approximately 60 min), PEG 1450/1000 mix at a ratio of 4:1 at 48ºC under vacuum until sunk (approximately 60 min), and embedded in PEG 1450/1000 mix at a ratio of 4:1. Sectioning was performed at room temperature on a rotary microtome to obtain 30 µm thick sections, which were placed 10 per well in PBS-azide (0.1%) and stored at 4ºC.

## Immunofluorescence Staining and Analysis

Cells cultured on chamber slides (BD Falcon) or glass coverslips were fixed with 4% paraformaldehyde (warmed to 37°C; Sigma-Aldrich) at room temperature for 20 min. For intracellular antigens, cells were permeabilized using 100% dimethylsulfoxide (DMSO; Wak-Chemie Medical GMBH, Germany) for 10 min, and then washed 3x with 0.1% Triton X-100 in PBS (used for all washes unless otherwise stated). Slides/coverslips were blocked with normal goat serum (10%) in PBS for 1 hour, followed by incubation with primary antibodies at room temperature for 1 hour. Primary antibodies included: mouse monoclonal antibodies (all IgG unless specified) against nestin (NES; 1:200; Chemicon), microtubule-associated protein-2 (MAP-2; 1:100; Sigma-Aldrich), neuronal nuclear antigen (NeuN; 1:200; Chemicon, Millipore, NSW, Australia), and tyrosine hydroxylase (TH; 1:1000; Immunostar Incorporated, WI, USA); and rabbit polyclonal antibodies against Fibronectin (1:400; Sigma-Aldrich), glial fibrillary acidic protein (GFAP; 1:200; Dako Cytomation, Glostrup, Denmark), and β tubulin III (1:2000; Covance, CA, USA). Slides/coverslips were washed 3x and incubated with Alexa Fluor 488 or 594 highly cross adsorbed goat anti-rabbit or anti-mouse secondary antibodies at 1:400 dilution for 1 hour. Negative controls were performed in all cases, consisting of secondary antibody application in the absence of primary antibody. Slide/coverslips were washed 3x, then nuclei were counterstained with 300 nM DAPI (Molecular Probes, Invitrogen) in PBS for 10-15 min, followed by a final PBS wash, and mounting in 100% glycerol (Bacto Laboratories, NSW, Australia). Cells were examined and imaged using a Zeiss inverted fluorescent microscope with Axiocam digital camera and Axiovision software (Carl Zeiss, Germany). For all neuronal differentiation procedures, immunofluorescence analysis was performed on 3 hMSC cultures, with control undifferentiated hMSCs from the same cultures examined in parallel.

Characterization of graft sites was performed on free-floating brain sections in 24 well plates using the same procedure described above with some modifications, including: all incubation and washing steps were performed with agitation on a shaker; washes were performed for 10 min; permeabilization with 100% DMSO for 20 min; overnight incubation with primary antibodies at 4ºC; and incubation with secondary antibodies for 3 hours. Additional primary antibodies used were rabbit anti-Iba-1 (1:2000; Wako Pure Chemical Industries Ltd (Osaka, Japan), mouse anti-GFAP-Cy3 (1:400; Sigma-Aldrich), and mouse anti-Human Nuclear Antigen (HNA; clone 235-1; MAB1281; 1:100; Chemicon, Millipore). Furthermore, the permeabilization step for HNA staining was performed with 0.1% Triton X-100 in PBS, rather than 100% DMSO, for 20 min.
