# Supplementary material for: Transplantation of Neuronal-Primed Human Bone Marrow Mesenchymal Stem Cells in Hemiparkinsonian Rodents
Source: PLoS One. 2011 May 23;6(5):e19025. doi: 10.1371/journal.pone.0019025 (PMC3100305; doi:10.1371/journal.pone.0019025)
Supplement: Table S1 — Primer pairs for real-time RT-PCR. (DOC) [file pone.0019025.s002.doc]

TABLE S1. Primer Pairs for Real-Time RT-PCR

| **Gene** | **GenBank Accession Number** | **Sequence (5'-3')**  **Sense Antisense** | | **Product Size (bp)** | **Positive Control** |
| --- | --- | --- | --- | --- | --- |
| **Pluripotency Markers** | | | | | |
| ***POU5F1*** | NM_002701 | ctcaccctgggggttctatt | agcttcctccacccacttct | 129 | hESCs |
| ***NANOG*** | NM_024865 | aactggccgaagaatagcaa | catccctggtggtaggaaga | 86 | hESCs |
| **Ectodermal Lineage Markers** | | | | | |
| ***NES*** | NM_006617 | tccaagacttccctcagctt | tcaggactgggagcaaagat | 145 | SK-N-SH |
| ***MAP-2*** | NM_002374 | agaccaccattgacgactcc | tctccgagcttccttttcag | 134 | SK-N-SH |
| ***CSPG4*** | NM_001897 | gaaggaggacggacctcaa | gatccatctcggaggcatta | 143 | U-87 MG |
| ***GFAP*** | NM_002055 | atcgagatcgccacctacag | caccacgatgttcctcttga | 150 | Brain |
| ***ENO2*** | NM_001975 | aggccagatcaagactggtg | caagcagaggaatcacagca | 148 | Brain |
| **Mesodermal Lineage Markers** | | | | | |
| ***COL1A1*** | NM_000088 | gagagcatgaccgatggatt | atgtaggccacgctgttctt | 149 | MG-63 |
| **Dopaminergic Neuronal Development Transcription Factors** | | | | | |
| ***NR4A2*** | NM_006186 | gctgttgggatggtcaaaga | ctgtgggctcttcggtttc | 87 | Brain |
| **Dopaminergic Neuronal Markers** | | | | | |
| ***TH*** | NM_000360 | cagccctaccaagaccagac | gtacgggtcgaacttcacg | 129 | Brain |
| **Ubiquitous Marker** | | | | | |
| ***HPRT1*** | NM_000194 | gaccagtcaacaggggacat | cctgaccaaggaaagcaaag | 132 |  |
| ***GAPDH*** | NM_002046 | aatcccatcaccatcttcca | tggactccacgacgtactca | 82 |  |

hESCs, human embryonic stem cells
